# Crystal structure of CdSO_4_(H_2_O): a redetermination

**DOI:** 10.1107/S2056989015016904

**Published:** 2015-09-17

**Authors:** Chatphorn Theppitak, Kittipong Chainok

**Affiliations:** a Department of Chemistry, Faculty of Science, Naresuan University, Muang, Phitsanulok, 65000, Thailand; b Department of Physics, Faculty of Science and Technology, Thammasat University, Khlong Luang, Pathum Thani, 12120, Thailand

**Keywords:** crystal structure, redetermination, cadmium sulfate monohydrate, hydro­thermal synthesis, hydrogen bonding

## Abstract

The crystal structure of the title compound, cadmium sulfate monohydrate or poly[(μ_2_-aqua)(μ_4_-sulfato)­cadmium], was redetermined based on modern CMOS (complementary metal oxide silicon) data. In comparison with the previous study [Bregeault & Herpin (1970 ▸). *Bull. Soc. Fr. Mineral. Cristallogr.* **93**, 37–42], all non-H atoms were refined with anisotropic displacement parameters and the hydrogen-bonding pattern unambiguously established due to location of the hydrogen atoms. In addition, a significant improvement in terms of precision and accuracy was achieved. In the crystal, the Cd^2+^ cation is coordinated by four O atoms of four sulfate anions and two O atoms of water mol­ecules, forming a distorted octa­hedral *trans*-[CdO_6_] polyhedron. Each sulfate anion bridges four Cd^2+^ cations and each water mol­ecule bridges two Cd^2+^ cations, leading to the formation of a three-dimensional framework, with Cd⋯Cd separations in the range 4.0757 (2)–6.4462 (3) Å. O—H⋯O hydrogen-bonding inter­actions of medium strength between the coordinating water mol­ecules and sulfate anions consolidate the crystal packing.

## Related literature   

For the previous report on the structure of the title compound, see: Bregeault & Herpin (1970 ▸).

## Experimental   

### Crystal data  

CdSO_4_(H_2_O)
*M* *_r_* = 226.48
Monoclinic, 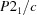

*a* = 7.6195 (3) Å
*b* = 7.4517 (3) Å
*c* = 8.1457 (3) Å
β = 122.244 (1)°
*V* = 391.17 (3) Å^3^
*Z* = 4
Mo *K*α radiation
μ = 6.01 mm^−1^
*T* = 296 K
0.26 × 0.22 × 0.22 mm

### Data collection  

Bruker APEXII D8 QUEST CMOS diffractometer
Absorption correction: multi-scan (*SADABS*; Bruker, 2014 ▸) *T*_min_ = 0.701, *T*_max_ = 0.746
17459 measured reflections
1004 independent reflections
958 reflections with *I* > 2σ(*I*)
*R*_int_ = 0.023

### Refinement  

*R*[*F* ^2^ > 2σ(*F* ^2^)] = 0.012
*wR*(*F* ^2^) = 0.026
*S* = 1.18
1004 reflections
72 parameters
2 restraints
All H-atom parameters refined
Δρ_max_ = 0.29 e Å^−3^
Δρ_min_ = −0.33 e Å^−3^

### 

Data collection: *APEX2* (Bruker, 2014 ▸); cell refinement: *SAINT* (Bruker, 2014 ▸); data reduction: *SAINT*; method used to solve structure: coordinates taken from previous refinement; program(s) used to refine structure: *SHELXL2014* (Sheldrick, 2015 ▸); molecular graphics: *OLEX2* (Dolomanov *et al.*, 2009 ▸); software used to prepare material for publication: *OLEX2* and *publCIF* (Westrip, 2010 ▸).

## Supplementary Material

Crystal structure: contains datablock(s) I. DOI: 10.1107/S2056989015016904/wm5211sup1.cif

Structure factors: contains datablock(s) I. DOI: 10.1107/S2056989015016904/wm5211Isup2.hkl

Supporting information file. DOI: 10.1107/S2056989015016904/wm5211Isup3.cdx

**Fig. 1.**
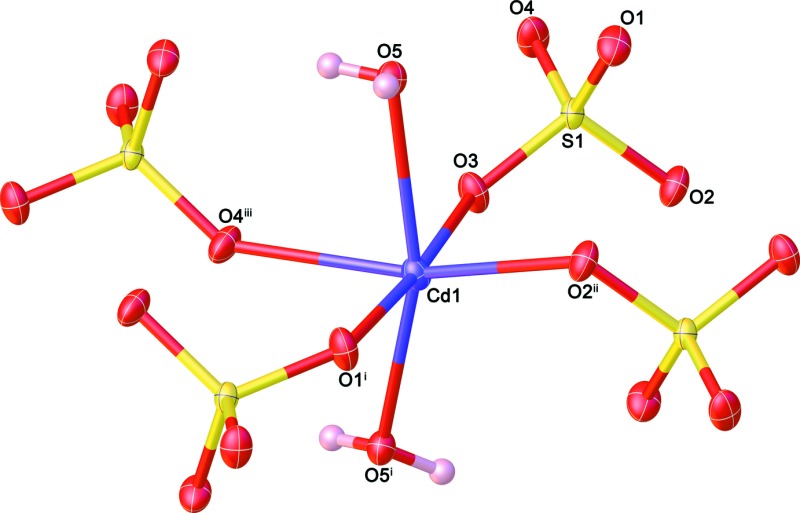
The coordination sphere around the Cd2+ cation with displacement ellipsoids drawn at the 50% probability level. [Symmetry codes: (i) x, 1/2 – y, 1/2 + z; (ii) –x, 1 – y, –z; (iii) 1 – x, -1/2 + y, 1/2 – z].

**Fig. 2.**
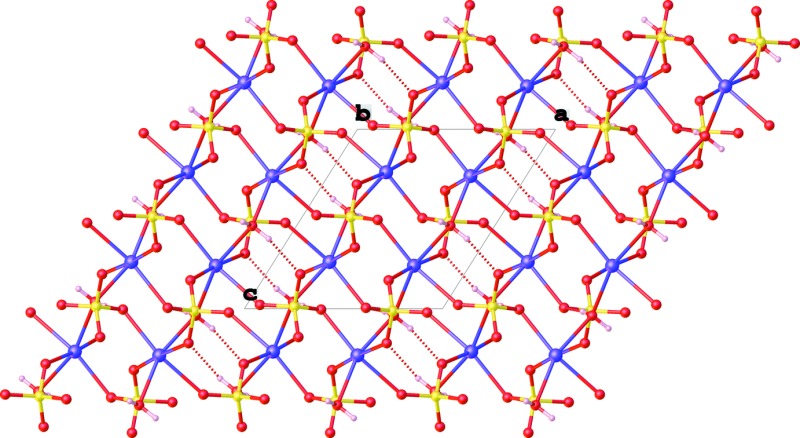
The three-dimensional framework structure of the title compound in a view along the b axis. Dashed lines indicate intermolecular O—H···O hydrogen-bonding interactions.

Click here for additional data file.2+ x y z x y z x y z . DOI: 10.1107/S2056989015016904/wm5211fig1.tif
The coordination sphere around the Cd^2+^ cation with displacement ellipsoids drawn at the 50% probability level. [Symmetry codes: (i) *x*, 1/2 – *y*, 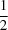 + *z*; (ii) –*x*, 1 – *y*, –*z*; (iii) 1 – *x*, −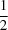 + *y*, 1/2 – *z*].

b . DOI: 10.1107/S2056989015016904/wm5211fig2.tif
The three-dimensional framework structure of the title compound in a view along the *b* axis. Dashed lines indicate inter­molecular O—H⋯O hydrogen-bonding inter­actions.

CCDC reference: 1423357

Additional supporting information:  crystallographic information; 3D view; checkCIF report

## Figures and Tables

**Table 1:** Hydrogen-bond geometry (, )

*D*H*A*	*D*H	H*A*	*D* *A*	*D*H*A*
O5H5*A*O3^i^	0.82(2)	1.88(2)	2.6958(17)	170(3)
O5H5*B*O2^ii^	0.86(2)	1.90(2)	2.7530(17)	173(2)

Symmetry codes: (i) 
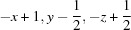
; (ii) 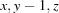.

**Table 2:** Comparison of bond lengths () in the current and the previous (Bregeault Herpin, 1970 ▸) refinement of cadmium sulfate monohydrate For the previous refinement: *a* = 7.607, *b* = 7.541, *c* = 8.186, = 121.86 and reliability index *R* = 0.12.

Bond	Current refinement	Previous refinement
Cd1O1^i^	2.2417(12)	2.21(5)
Cd1O2^ii^	2.2530(13)	2.27(3)
Cd1O3	2.2421(12)	2.36(5)
Cd1O4^iii^	2.3112(12)	2.33(3)
Cd1O5^i^	2.3210(12)	2.24(3)
Cd1O5	2.4024(12)	2.33(3)
S1O1	1.4703(12)	1.50(4)
S1O2	1.4845(12)	1.62(6)
S1O3	1.4831(12)	1.45(3)
S1O4	1.4584(12)	1.42(4)

Symmetry codes: (i) *x*, *y*+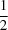, *z*+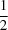; (ii) *x*, *y*+1, *z*; (iii) *x*+1, *y* 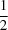, *z*+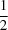.

## supplementary crystallographic information

### S1. Synthesis and crystallization

The title compound was obtained serendipitously. CdSO_4_·8/3H_2_O (0.256 g, 1 mmol) and 3,6-di-2-pyridyl-1,2,4,5-tetra­zine (0.236 g, 1 mmol) dissolved in water (5 ml) were added to a 23 ml Teflon-lined autoclave and heated at 356 K for 5 days. The product was collected by filtration, washed with water and air-dried. Colourless block-shaped crystals of the title compound suitable for X-ray analysis were isolated.

### S2. Refinement

The same cell setting and atom numbering scheme as in the previous refinement (Bregeault & Herpin, 1970) were used. Starting coordinates for the atoms were also taken from the previous model. Hydrogen atoms of the water molecules were located from difference Fourier maps and were refined with an O—H distance restraint of 0.85 (2) Å.

### Crystal data

**Table:**

CdSO_4_(H_2_O)	*F*(000) = 424
*M**_r_* = 226.48	*D*_x_ = 3.846 Mg m^−^^3^
Monoclinic, *P*2_1_/*c*	Mo *K*α radiation, λ = 0.71073 Å
*a* = 7.6195 (3) Å	Cell parameters from 9894 reflections
*b* = 7.4517 (3) Å	θ = 3.2–30.5°
*c* = 8.1457 (3) Å	µ = 6.01 mm^−^^1^
β = 122.244 (1)°	*T* = 296 K
*V* = 391.17 (3) Å^3^	Block, colourless
*Z* = 4	0.26 × 0.22 × 0.22 mm

### Data collection

**Table:**

Bruker APEXII D8 QUEST CMOS diffractometer	1004 independent reflections
Radiation source: microfocus sealed x-ray tube, Incoatec Iµus	958 reflections with *I* > 2σ(*I*)
GraphiteDouble Bounce Multilayer Mirror monochromator	*R*_int_ = 0.023
Detector resolution: 10.5 pixels mm^-1^	θ_max_ = 28.7°, θ_min_ = 3.2°
ω and φ scans	*h* = −10→10
Absorption correction: multi-scan (*SADABS*; Bruker, 2014)	*k* = −10→10
*T*_min_ = 0.701, *T*_max_ = 0.746	*l* = −10→10
17459 measured reflections	

### Refinement

**Table:**

Refinement on *F*^2^	Primary atom site location: structure-invariant direct methods
Least-squares matrix: full	Hydrogen site location: difference Fourier map
*R*[*F*^2^ > 2σ(*F*^2^)] = 0.012	All H-atom parameters refined
*wR*(*F*^2^) = 0.026	*w* = 1/[σ^2^(*F*_o_^2^) + (0.0107*P*)^2^ + 0.2955*P*] where *P* = (*F*_o_^2^ + 2*F*_c_^2^)/3
*S* = 1.18	(Δ/σ)_max_ = 0.001
1004 reflections	Δρ_max_ = 0.28 e Å^−^^3^
72 parameters	Δρ_min_ = −0.33 e Å^−^^3^
2 restraints	

### Special details

**Table:**

Experimental. SADABS was used for absorption correction. wR2(int) was 0.0449 before and 0.0357 after correction. The Ratio of minimum to maximum transmission is 0.9396. The λ/2 correction factor is 0.00150.
Geometry. All e.s.d.'s (except the e.s.d. in the dihedral angle between two l.s. planes) are estimated using the full covariance matrix. The cell e.s.d.'s are taken into account individually in the estimation of e.s.d.'s in distances, angles and torsion angles; correlations between e.s.d.'s in cell parameters are only used when they are defined by crystal symmetry. An approximate (isotropic) treatment of cell e.s.d.'s is used for estimating e.s.d.'s involving l.s. planes.

### Fractional atomic coordinates and isotropic or equivalent isotropic displacement parameters (Å^2^)

**Table:**

	*x*	*y*	*z*	*U*_iso_*/*U*_eq_	
Cd1	0.21887 (2)	0.26013 (2)	0.26053 (2)	0.01330 (5)	
S1	0.25524 (6)	0.61729 (5)	0.01217 (5)	0.01018 (8)	
O1	0.13754 (18)	0.50022 (16)	−0.15811 (17)	0.0183 (2)	
O2	0.1103 (2)	0.75977 (15)	−0.00480 (19)	0.0169 (3)	
O3	0.32756 (18)	0.51255 (16)	0.19211 (17)	0.0168 (2)	
O4	0.43393 (18)	0.69973 (18)	0.02148 (18)	0.0184 (2)	
O5	0.27603 (19)	0.09752 (16)	0.03844 (17)	0.0140 (2)	
H5A	0.400 (3)	0.074 (4)	0.110 (3)	0.038 (7)*	
H5B	0.216 (3)	−0.005 (2)	0.016 (3)	0.023 (6)*	

### Atomic displacement parameters (Å^2^)

**Table:**

	*U*^11^	*U*^22^	*U*^33^	*U*^12^	*U*^13^	*U*^23^
Cd1	0.01176 (7)	0.01373 (7)	0.01200 (7)	−0.00165 (4)	0.00472 (5)	0.00087 (4)
S1	0.00809 (16)	0.00898 (17)	0.01063 (17)	0.00001 (13)	0.00310 (14)	0.00041 (13)
O1	0.0156 (6)	0.0170 (6)	0.0158 (6)	−0.0004 (5)	0.0041 (5)	−0.0054 (5)
O2	0.0122 (6)	0.0134 (6)	0.0206 (6)	0.0027 (4)	0.0058 (5)	−0.0018 (4)
O3	0.0155 (6)	0.0153 (6)	0.0147 (5)	−0.0019 (5)	0.0048 (5)	0.0046 (4)
O4	0.0101 (5)	0.0224 (6)	0.0198 (6)	−0.0022 (5)	0.0061 (5)	0.0047 (5)
O5	0.0120 (5)	0.0133 (5)	0.0145 (5)	−0.0008 (4)	0.0055 (5)	−0.0001 (4)

### Geometric parameters (Å, º)

**Table:**

Cd1—O1^i^	2.2417 (12)	S1—O3	1.4831 (12)
Cd1—O2^ii^	2.2530 (13)	S1—O4	1.4584 (12)
Cd1—O3	2.2421 (12)	O1—Cd1^iv^	2.2417 (12)
Cd1—O4^iii^	2.3112 (12)	O2—Cd1^ii^	2.2530 (13)
Cd1—O5^i^	2.3210 (12)	O4—Cd1^v^	2.3112 (12)
Cd1—O5	2.4024 (12)	O5—Cd1^iv^	2.3211 (12)
S1—O1	1.4703 (12)	O5—H5A	0.822 (17)
S1—O2	1.4845 (12)	O5—H5B	0.859 (16)
			
O1^i^—Cd1—O2^ii^	82.50 (4)	O1—S1—O3	109.76 (7)
O1^i^—Cd1—O3	175.24 (4)	O3—S1—O2	109.52 (8)
O1^i^—Cd1—O4^iii^	89.31 (5)	O4—S1—O1	112.43 (8)
O1^i^—Cd1—O5^i^	92.57 (4)	O4—S1—O2	109.35 (7)
O1^i^—Cd1—O5	88.62 (4)	O4—S1—O3	109.03 (7)
O2^ii^—Cd1—O4^iii^	161.94 (4)	S1—O1—Cd1^iv^	131.81 (7)
O2^ii^—Cd1—O5	80.22 (4)	S1—O2—Cd1^ii^	116.42 (7)
O2^ii^—Cd1—O5^i^	117.97 (4)	S1—O3—Cd1	134.06 (7)
O3—Cd1—O2^ii^	101.65 (4)	S1—O4—Cd1^v^	140.91 (8)
O3—Cd1—O4^iii^	86.05 (4)	Cd1^iv^—O5—Cd1	119.27 (5)
O3—Cd1—O5	89.80 (4)	Cd1^iv^—O5—H5A	109.7 (18)
O3—Cd1—O5^i^	87.54 (4)	Cd1—O5—H5A	99.9 (18)
O4^iii^—Cd1—O5^i^	78.31 (4)	Cd1^iv^—O5—H5B	113.3 (14)
O4^iii^—Cd1—O5	83.53 (4)	Cd1—O5—H5B	108.6 (14)
O5^i^—Cd1—O5	161.78 (6)	H5A—O5—H5B	104 (2)
O1—S1—O2	106.70 (7)		
			
O1—S1—O2—Cd1^ii^	2.46 (10)	O3—S1—O1—Cd1^iv^	−54.81 (11)
O1—S1—O3—Cd1	−23.82 (12)	O3—S1—O2—Cd1^ii^	−116.29 (8)
O1—S1—O4—Cd1^v^	−135.57 (12)	O3—S1—O4—Cd1^v^	−13.61 (15)
O2—S1—O1—Cd1^iv^	−173.39 (9)	O4—S1—O1—Cd1^iv^	66.74 (12)
O2—S1—O3—Cd1	93.01 (11)	O4—S1—O2—Cd1^ii^	124.30 (8)
O2—S1—O4—Cd1^v^	106.10 (13)	O4—S1—O3—Cd1	−147.38 (10)

Symmetry codes: (i) *x*, −*y*+1/2, *z*+1/2; (ii) −*x*, −*y*+1, −*z*; (iii) −*x*+1, *y*−1/2, −*z*+1/2; (iv) *x*, −*y*+1/2, *z*−1/2; (v) −*x*+1, *y*+1/2, −*z*+1/2.

### Hydrogen-bond geometry (Å, º)

**Table:**

*D*—H···*A*	*D*—H	H···*A*	*D*···*A*	*D*—H···*A*
O5—H5*A*···O3^iii^	0.82 (2)	1.88 (2)	2.6958 (17)	170 (3)
O5—H5*B*···O2^vi^	0.86 (2)	1.90 (2)	2.7530 (17)	173 (2)

Symmetry codes: (iii) −*x*+1, *y*−1/2, −*z*+1/2; (vi) *x*, *y*−1, *z*.

## References

[bb1] Bregeault, J. M. & Herpin, P. (1970). *Bull. Soc. Fr. Mineral. Cristallogr.* **93**, 37–42.

[bb2] Bruker (2014). *APEX2*, *SADABS* and *SAINT*. Bruker AXS Inc., Madison, Wisconsin, USA.

[bb3] Dolomanov, O. V., Bourhis, L. J., Gildea, R. J., Howard, J. A. K. & Puschmann, H. (2009). *J. Appl. Cryst.* **42**, 339–341.

[bb4] Sheldrick, G. M. (2015). *Acta Cryst.* C**71**, 3–8.

[bb5] Westrip, S. P. (2010). *J. Appl. Cryst.* **43**, 920–925.

