# Supplementary figures and images for: Crystal structure of CdSO4(H2O): a redetermination
Source: Acta Crystallogr E Crystallogr Commun. 2015 Sep 17;71(Pt 10):i8–9. doi: 10.1107/S2056989015016904 (PMC4647421; doi:10.1107/S2056989015016904)

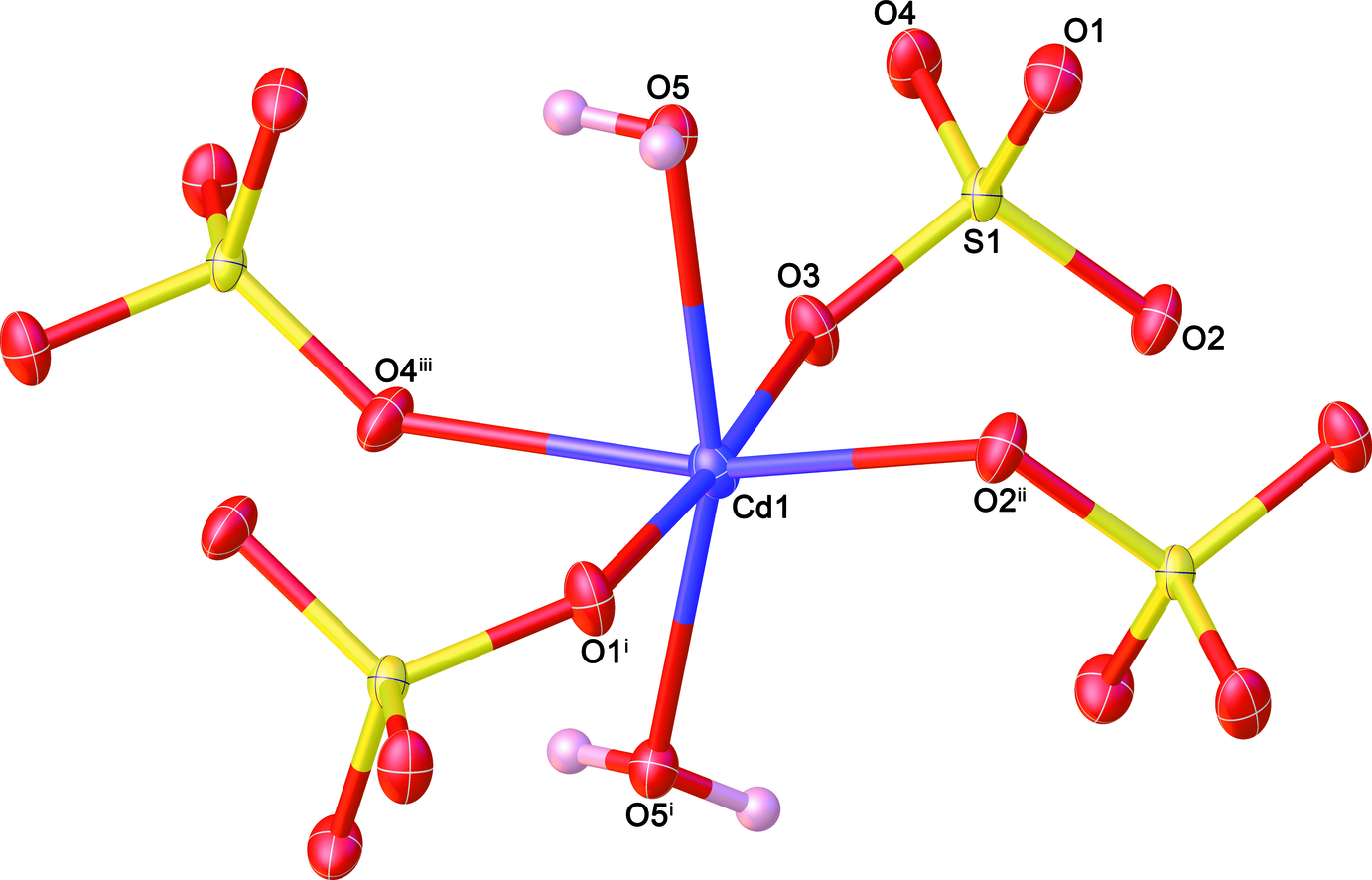

Supplement: Supplementary file 4 [file e-71-000i8-fig1.tif]

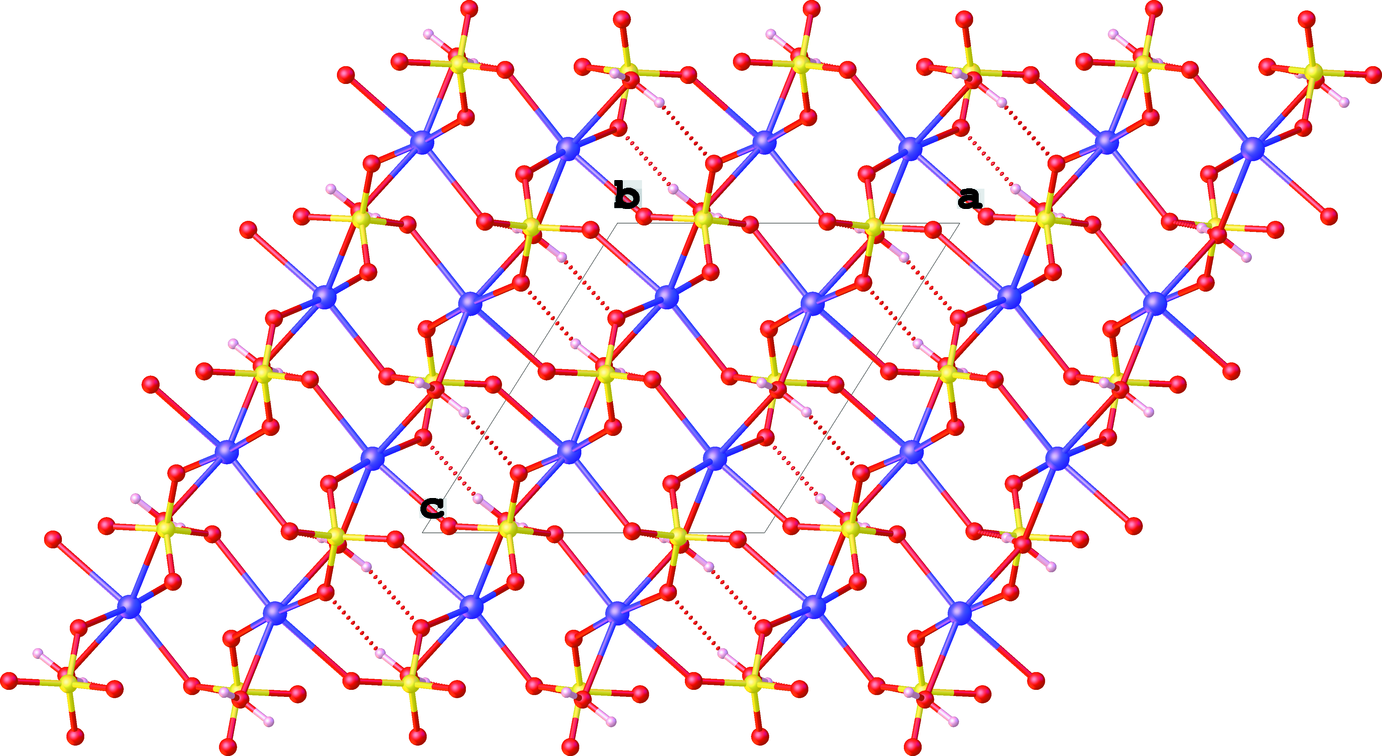

Supplement: Supplementary file 5 [file e-71-000i8-fig2.tif]
